# Prognostic models for outcome prediction following in-hospital cardiac arrest using pre-arrest factors: a systematic review, meta-analysis and critical appraisal

**DOI:** 10.1186/s13054-023-04306-y

**Published:** 2023-01-20

**Authors:** Casey Grandbois van Ravenhorst, Marc Schluep, Henrik Endeman, Robert-Jan Stolker, Sanne Elisabeth Hoeks

**Affiliations:** 1 Department of Anaesthesia, Erasmus University Medical Centre, Room Na-1718, P.O. Box 2040, 3000 CA Rotterdam, The Netherlands; 2 Department of Intensive Care Medicine, Erasmus University Medical Centre, Rotterdam, The Netherlands

**Keywords:** In-hospital cardiac arrest, Cardiopulmonary resuscitation, Clinical outcome, Prognostic model

## Abstract

**Background:**

Several prediction models of survival after in-hospital cardiac arrest (IHCA) have been published, but no overview of model performance and external validation exists. We performed a systematic review of the available prognostic models for outcome prediction of attempted resuscitation for IHCA using pre-arrest factors to enhance clinical decision-making through improved outcome prediction.

**Methods:**

This systematic review followed the CHARMS and PRISMA guidelines. Medline, Embase, Web of Science were searched up to October 2021. Studies developing, updating or validating a prediction model with pre-arrest factors for any potential clinical outcome of attempted resuscitation for IHCA were included. Studies were appraised critically according to the PROBAST checklist. A random-effects meta-analysis was performed to pool AUROC values of externally validated models.

**Results:**

Out of 2678 initial articles screened, 33 studies were included in this systematic review: 16 model development studies, 5 model updating studies and 12 model validation studies. The most frequently included pre-arrest factors included age, functional status, (metastatic) malignancy, heart disease, cerebrovascular events, respiratory, renal or hepatic insufficiency, hypotension and sepsis. Only six of the developed models have been independently validated in external populations. The GO-FAR score showed the best performance with a pooled AUROC of 0.78 (95% CI 0.69–0.85), versus 0.59 (95%CI 0.50–0.68) for the PAM and 0.62 (95% CI 0.49–0.74) for the PAR.

**Conclusions:**

Several prognostic models for clinical outcome after attempted resuscitation for IHCA have been published. Most have a moderate risk of bias and have not been validated externally. The GO-FAR score showed the most acceptable performance. Future research should focus on updating existing models for use in clinical settings, specifically pre-arrest counselling.

*Systematic review registration* PROSPERO CRD42021269235. Registered 21 July 2021.

**Supplementary Information:**

The online version contains supplementary material available at 10.1186/s13054-023-04306-y.

## Background

Advance care directives are becoming increasingly important in modern day medical practice. The possibility of successful cardiopulmonary resuscitation (CPR) in case of cardiac arrest is the quintessential directive to discuss. Expected prognosis after attempted CPR for in-hospital cardiac arrest (IHCA) is an increasingly important part of the dialogue. Providing adequate guidance can be challenging, especially as patients tend to overestimate their likelihood of survival [1]. Even though the likelihood of survival and the chance at good neurological outcome after IHCA remains poor [2]. Ideally, clinicians would be able to identify both patients who have a good chance at qualitative survival after cardiopulmonary resuscitation, as well as patients with a low chance of survival, in whom futile resuscitation attempts could be avoided.

Compared to out-of-hospital cardiac arrest (OHCA), there is limited data on outcome after IHCA [3]. Although evidence from OHCA is often extrapolated to IHCA, the epidemiology is different and the determinants of survival and outcome differ accordingly [4]. There is a need for prognostication tools to guide clinicians in decision-making and counselling of patients regarding IHCA. Although several significant peri-arrest prognostic factors for IHCA have been identified, patients and clinicians must rely on pre-arrest factors to establish a CPR-directive [5].

Several risk models were published over the years addressing this clinical dilemma. However, there is still little evidence supporting clinical decision-making [4] and no model has up to now been implemented in clinical practice. An overview of the developed prognostic tools has recently been published, however the focus lay on establishing diagnostic accuracy [6]. The aim of this study was to summarize and appraise prediction models for any clinical outcome after attempted CPR for IHCA using pre-arrest variables, to assess the extent of validation in external populations, and to perform a meta-analysis of the performance of the prognostic models. Clinicians could thus improve the prediction of outcome after IHCA in order to better inform their patients and enhance clinical decision-making.

## Methods

This systematic review was designed according to the Checklist for critical Appraisal and data extraction for systematic Reviews of prediction Modelling Studies (CHARMS) and the guidance as described by Debray et al. [7, 8] A protocol was registered in the International Prospective Register of Systematic Reviews PROSPERO (CRD42021269235). Data reporting and review are consistent with the Preferred Reporting Items for Systematic Reviews and Meta-Analyses (PRISMA) statement [9]. The review question was formulated using the PICOTS scheme (Population, Intervention, Comparator, Outcome, Timing and Setting) (Additional file 1: Appendix, Table S1).

### Literature search

A systematic search in MEDLINE was performed via PubMed, Embase and the Cochrane Library for studies published from inception to 22-10-2021. An experienced librarian assisted in developing the search strategy, which included synonyms for [in-hospital cardiac arrest], combined with [prognostic model/prognosis/prediction/outcome assessment]. (Additional file 1: Appendix) The recommendations by Geersing et al. [10] regarding search filters specifically for finding prediction model studies for systematic reviews were followed, as well as those by Bramer et al. using single paragraph searches [11]. Two authors (CGvR, MS) independently screened titles/abstracts and full text articles and discrepancies were resolved by a third author (SH). References of each eligible article were hand searched for potential further inclusion.

### Selection criteria

Studies specifically developing, validating and/or updating a multivariable prognostic model for any clinical outcome after attempted resuscitation for IHCA were included. A study was considered eligible following the definition of prognostic model studies as proposed by the Transparent Reporting of a multivariable prediction models for Individual Prognosis Or Diagnosis (TRIPOD) statement [12]. Eligible studies should specifically report the development, update or recalibration, or external validation of prognostic models to predict outcome after in-hospital cardiac arrest using pre-arrest factors and report model performance measures. No language restrictions were imposed.

### Outcome assessment

Eligible outcomes were any possible clinical outcome after IHCA, such as the return of spontaneous circulation (ROSC), survival to discharge (or longer term survival) and neurological outcome (Cognitive Performance Category: CPC). Studies only including peri-arrest factors were excluded, as these prognostic factors are not available at the time of advance care planning. Studies exclusively describing data of patients after ROSC or studies of mixed OHCA/IHCA populations without separate reporting for IHCA-patients were also excluded.

### Definitions and terminology

A prognostic model was defined as ‘a formal combination of multiple prognostic factors from which risks of a specific end point can be calculated for individual patients’ [13]. A good clinical prediction model should discriminate between patients who do and do not experience a specific event (discrimination), make accurate predictions (calibration) and perform well across different patient populations (generalisability) [14, 15]. Discrimination is often expressed by the concordance statistic (C-statistic)—the chance that a randomly selected patient who experiences an event has a higher score in the model than a random patient who does not. For binary outcomes, the C-statistic is equal to the area under the operating receiver curve (AUC). Calibration compares the predicted probability of survival with actual survival [16]. It is often visualised with a calibration plot and/ or goodness-of-fit (GOF) as quantified by the Hosmer–Lemeshow test. Other measures of model performance are sensitivity, specificity, positive and negative predictive value, accuracy, *R*^2^-statistic and Brier score.

### Data extraction

A standardised form following the CHARMS checklist was developed in which two authors independently extracted data (CGvR, MS) [7]. Articles were categorised into development, updating/recalibration and validation subgroups. For all eligible articles, the following information was extracted: first author and year of publication, model name, study population, sample size, source of data (i.e. study design, date of enrolment), number of centres, countries of inclusion, predicted outcome, factors in the model, model performance and information on validation. For development/update studies, model development method, number of prognostic factors screened and final model presentation were collected. Separate individual prognostic factors of the models were tabulated.

### Statistical analysis

For prediction models that had been externally validated in multiple studies, a random-effect meta-analysis was performed of the reported AUC’s to yield a pooled AUC for each prediction model [8]. 95% confidence intervals (CI) and (approximate) 95% prediction intervals (PI) were calculated to quantify uncertainty and the presence of between-study heterogeneity. Analyses were performed in R version 4.2.1 using the package metamisc.

### Quality assessment

The Prediction model Risk Of Bias Assessment Tool (PROBAST) was used to apply the risk of bias assessment of the studies developing or validating prognostic models [17]. Assessment of methodological quality was done separately by two authors (CGvR, MS).

## Results

**Fig. 1:**
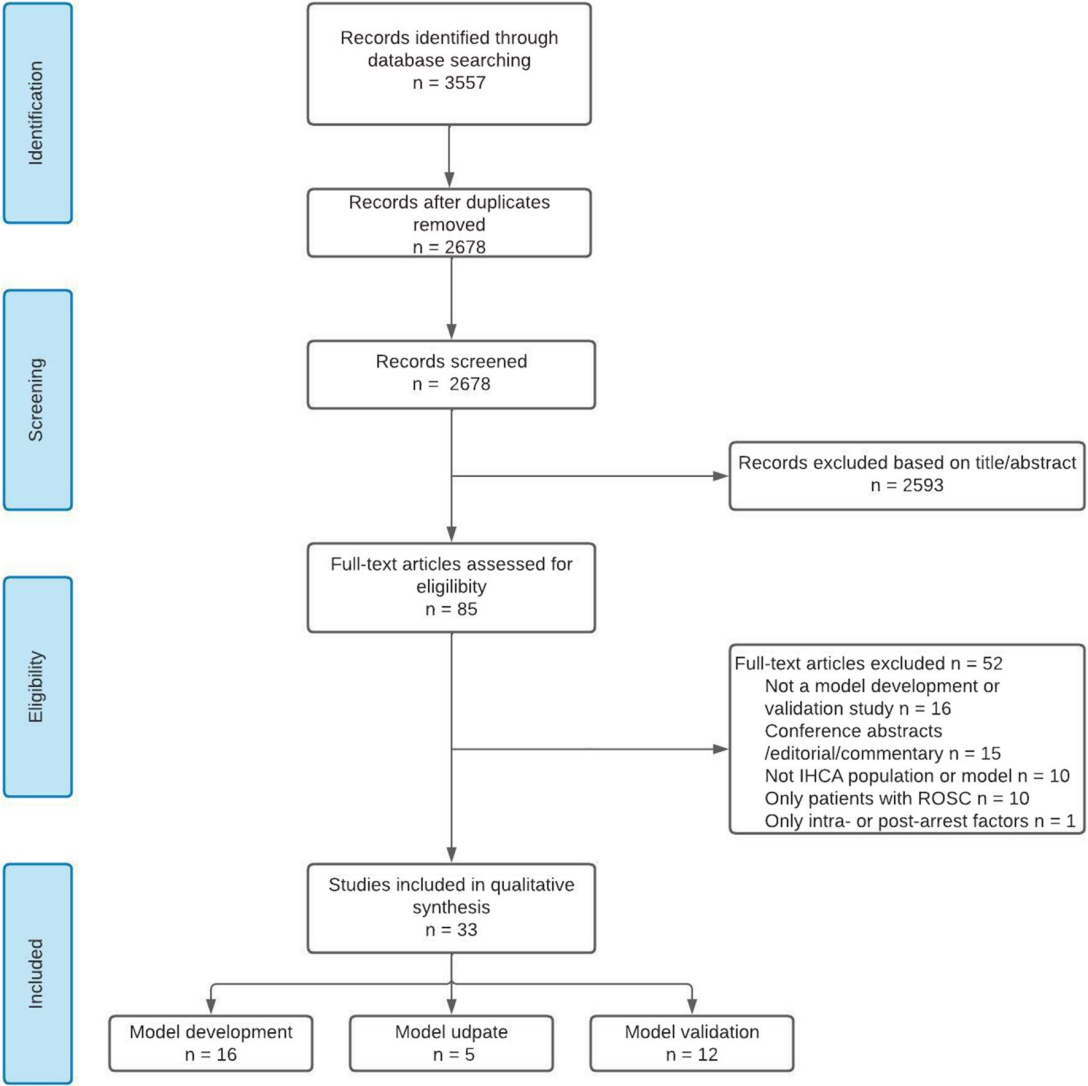
Flow diagram of literature search and included studies

A total of 2678 studies were screened (Fig. 1). Flow diagram of literature search and included studies.) and 33 studies were included in the qualitative synthesis of this systematic review: 16 model development studies [18–33], five model updating studies [34–38] and 12 model validation studies [39–50] (Tables 1, 2 and 4). All studies included patients that received CPR for IHCA. In five studies [20, 21, 25, 26, 31], multiple models were developed resulting in a total of 22 developed models in 16 studies. Of these, seven studies reported (internal) validation of the developed model and three of the five model updating studies reported validation in the original paper (Table 2). Most models were developed or updated using registries as source of data (9/21 studies) or data from retrospective cohorts (7/21 studies). Three studies used a prospective cohort, and in two studies, the source of data was not mentioned (Table 1).

**Table 1:** Model development and updating studies

Reference (first author, year of publication)	Model	Study population	Predicted outcome	No. of participants (no. with predicted outcome)	Study interval	Source of data	Country of inclusion (no. of centres)	Model development	No. of variables screened (no. In final model)
*Model development*									
Swindell et al. [18]	ABCD-score	Attempted CPR for IHCA in patients > 50 years	Survival to discharge	92.706 (n.r.)	2012–2016	NIS Registry	USA (n.r.)	Selection of variables based on univariate analysis	n.r. (4)
Chan et al. [19]	Benchmark hospital rates for ROSC after IHCA	Attempted CPR for IHCA	Sustained ROSC > 20 min	83.206 (D: 55.601 (40.038), V:27.128 (19.716))	2014–2017	GWTG Registry	USA (335)	Hierarchical logistic regression	24 (10)
Harrison et al. [20]	1. Predicting ROSC following IHCA	Attempted CPR for IHCA	ROSC > 20 min	22.479 (D: 14.688 (6605), V: 7791 (2926))	2011–2013	NCAA Registry	UK (143)	Multilevel logistic regression	7 (7)
	2. Predicting survival following IHCA	Attempted CPR for IHCA	Survival to discharge						7 (6)
Ebell et al. [22]#	1. Classification and Regression Trees (CART1)	Attempted CPR for IHCA	Survival to discharge with CPC = 1	52.527 D: 38.092 (3610) V: 14.435 (1720)	2007–2009	GWTG Registry	USA (366)	Classification and regression trees	32 (8)
	2. Classification and Regression Trees (CART2)								32 (9)
Ebell et al. [21]	GO-FAR	Attempted CPR for IHCA	Survival to discharge with CPC = 1	51.240: training (44%), testing (22%), validation (34%) (overall 10.4% with outcome)	2007–2009	GWTG Registry	USA (366)	Previous meta-analysis; logistic regression	17 (13)
Chan et al. [23]	Risk-standardized survival after IHCA	Attempted CPR for IHCA	Survival to hospital discharge; risk-standardized survival rates	48.841 (10.290) (D: 32.560 (6844), V:16.281 (3446))	2007–2010	GWTG Registry	USA (356)	Hierarchical logistic regression	26 (9)
Larkin et al. [24]	NRCPR in-hospital mortality prediction model	Attempted CPR for IHCA	In-hospital mortality (or CPC ≥ 3)	49,130 (84.1% with outcome) (D: 33,468 V: 12,754)	2000–2004	NRCPR Registry	USA (366)	Logistic regression with bootstrapping	63 (33)
Danciu et al. [25]	1. Survival after IHCA	Attempted CPR for IHCA	Survival to discharge	219 (33)	2000–2002	Retrospective cohort	USA (1)	Backwards stepwise logistic regression	10 (5)
	2. Survival after IHCA		1 month survival	219 (30)					
	3. Survival after IHCA		3 months survival	219 (25)					
Cooper et al. [26]**	Resuscitation Predictor Scoring Scale (RPS Scale)	Attempted CPR for IHCA	24-h survival	1633 (444)	1993–2000	Retrospective cohort	UK (1)	Multiple forward logistic regression	n.r. (3)
Ambery et al. [27]	IHCA 5-score	Attempted CPR for IHCA	Survival to discharge	111 (14)	1998–1999	Retrospective cohort	UK (1)	Previous knowledge	5 (5)
Dodek et al. [28]	Predictors of in-hospital mortality after IHCA	Attempted CPR for IHCA	Probability of death before hospital discharge	D: 197 (145) V:120 (90)	1986–87; 1989–90	Retrospective cohort	Canada (1)	Stepwise logistic regression	31 (8)
Ebell et al. [29]	Artificial neural networks for predicting failure to survive following IHCA	Attempted CPR for IHCA (ward + ICU)	Failure to survive	218 (96)	1989–1991	Retrospective cohort	USA (1)	Artificial neural network	24 (24)
Lawrence et al. [30]	Probability of survival after CPR for IHCA	Attempted CPR for IHCA	Survival to discharge	340 (88)	1987–1989	Retrospective cohort	USA (1)	Stepwise discriminant analysis followed by logistic regression	18 (4)
Marwick et al. [31]**	1. Prognostic survival index at resuscitation attempt	Attempted CPR for IHCA (ward + CCU + ER + OR)	Survival to discharge	710 (92)	1984–1987	Prospective cohort	Australia (1)	Cox multiple regression	8 (4)
	2.Prognostic survival index after 10 min								8 (3)
	3.Prognostic survival index after resuscitation								8 (5)
George et al. [32]	Pre-Arrest Morbidity Index (PAM)	Attempted closed-chest CPR for IHCA (ward + ICU)	ROSC, survival to discharge, 3-months survival	140 (77, 34, 29)	1985	Prospective cohort	USA (1)	Previous knowledge	n.r. (15)
Burns et al. [33]	Prediction of initial success after CPR for IHCA	Attempted CPR for IHCA (ward + ICU)	ROSC	122 (56)	1985	Retrospective cohort	USA (1)	Significance testing followed by backwards logistic regression	25 (4)

Model updating										Model based on
Hong et al. [34]	GO-FAR + albumin	Attempted CPR for IHCA	Survival to discharge with CPC 1 or 2	863 (127): D 419 (59) and V: 444 (68)	2013–2017	Prospective cohort	Korea (1)	Previous knowledge	14 (14)	GO-FAR
George et al. [35]	GO-FAR 2	Attempted CPR for IHCA (excl. OR)	Survival to discharge with CPC ≤ 2	52.468: training 23.713 (44%) (4768 with outcome), testing 12.240 (22%), validation 16.515 (34%)	2012–2017	GWTG Registry	USA (366)	Previous knowledge; BIC and LASSO analysis	24 (10)	GO-FAR
Piscator et al. [36]	Prediction of outcome for In-Hospital Cardiac Arrest (PIHCA)	Attempted CPR for IHCA	Survival to discharge with CPC ≤ 2	717 (628 after exclusion missing cases) (174)	2013–2014	SCR Registry	Sweden (6)	Previous knowledge; logistic regression	9 (9)	GO-FAR
Dautzenberg et al. [37]*	Modified PAM index (MPI)	n.r. (according to George et al.)	n.r. (according to George et al.)	n.r.	n.r.	n.r.	n.r.	Previous knowledge	n.r. (16)	PAM
Ebell et al. [38]*	Prognosis After Resuscitation (PAR)	n.r. (according to George et al.)	n.r. (according to George et al.)	218 (n.r.)	n.r.	n.r.	n.r.	Previous knowledge	n.r. (8)	PAM

*D* Derivation, *V* Validation, *n.r.* not reported

*Studies do not formally fit the inclusion criteria due to lack of reporting model performance measures. They were nevertheless included as they were validated in external populations. They validated the scores for different end points but no formal process of model development has taken place

**Only age as pre-arrest variable

^#^Five models are reported to be made but only the best two are presented, performance of all five is reported

**Table 2:** Model characteristics of model development and updating studies

Reference (first author, year of publication)	Type of factors in final model	Prognostic factors in final model	Model presentation	Evaluation of model performance			Validation
				Calibration	Discrimination	Classification	
*Model development*							
Swindell et al. [18]	Pre + intra-arrest	Age, BMI, comorbidity (cancer or liver disease), days from admission to arrest	Scoring system	n.r.	AUC 0.581 (95% CI 0.577–0.585)	ABDC ≤ 2 sensitivity 96.8% (accuracy 33.6%), ABCD ≤ -1 specificity 82%	n.r.
Chan et al. [19]	Pre + intra-arrest	Age, initial rhythm, heart failure during admission, respiratory insufficiency, diabetes mellitus, metabolic disturbance, metastatic/haematologic malignancy, acute CNS non-stroke event, continuous IV vasopressor, mechanical ventilation	Logistic regression model with beta-coefficients estimates	*D*:* R*^2^ = 0.996 *V*:* R*^2^ = 0.990, Hosmer-Lemeshow GOF = 0.87	*D*: c-statistic 0.638 *V*: c-statistic 0.630	n.r.	Internal validation: split-sample
Harrison et al. [20]	Pre + intra-arrest	1. ROSC: age, sex, prior length of stay, reason for attendance, location of arrest, initial rhythm, interactions presenting rhythm and location	Online score calculator	Calibration plots	1.* D*: c-statistic = 0.727 V: c-index 0.73. Accuracy:* R*^2^ = 0.11–0.17	n.r.	External validation: temporal validation, geographical validation
		2. Survival: age, prior length of stay, reason for attendance, location of arrest, initial rhythm, interactions presenting rhythm and location			2.* D*: c-statistic = 0.804. * V*: c-index 0.81. Accuracy:* R*^2^ = 0.21–0.24		
Ebell et al. [21]	Pre-arrest	1: CPC-score, admitting location, sepsis, mechanical ventilation, age, metastatic/haematological malignancy, acute MI this admission	Algorithm	n.r.	AUC: Model 1* D*: 0.76,* V*: 0.73	Classification table	External validation: temporal validation
		2: Factors of model 1 + other configuration + acute stroke			AUC: Model 2* D*: 0.74,* V*: 0.71		
Ebell et al. [21]	Pre-arrest	Age, admission neurologically intact, major trauma, acute stroke, metastatic/haematological cancer, septicaemia, non-cardiac admission, hypotension/hypoperfusion, respiratory insufficiency, pneumonia, metastatic cancer, renal dialysis/insufficiency, hepatic insufficiency, admitted from nursing facility	Scoring system	Hosmer–Lemeshow statistic 11.39 (*p* = 0.18). HL graph is shown	C-statistic overall: 0.800. Derivation and training: 0.77, validation 0.78	Classification table	Internal validation: split-sample
Chan et al. [23]	Pre + intra	Age, initial arrest rhythm, hospital location, hypotension, septicemia, metastatic/haematological malignancy, hepatic insufficiency, mechanical ventilation before arrest, vasopressor before arrest	Logistic regression model with beta-coefficients estimates	*R*^2^ of 0.99 in derivation and validation cohorts	*C*-statistic: 0.734 in derivation and 0.737 in validation cohort	n.r.	Internal validation: split-sample
Larkin et al. [24]	Pre + intra-arrest	Code team present, age, race, illness category (medical cardiac, surgical cardiac, surgical non-cardiac and trauma, obstetrics), pre-existing conditions (MI, hypotension, hepatic insufficiency, baseline depression CNS function, acute stroke, infection/septicemia, metastatic/haematological malignancy, renal failure, major trauma), interventions in place (invasive airway, mechanical ventilation, anti-arrhythmics, vasopressors, vasodilators, chest tube), monitored outside ICU, monitored with arterial catheter, witnessed, pulseless when need for CPR recognized, event location (PACU/OR, general floor/telemetry, ED), initial pulseless rhythm (asystole vs VF, VT), admit time to event (in h)	Multivariate odds ratios	*D*: Hosmer-Lemeshow statistic 73.7 (*p* < 0.001) V: Hosmer-Lemeshow statistic 37.9 (*p* < 0.001)	*D*: AUC 0.78.* V*: AUC 0.77 (0.79–0.78). For data known at admission: AUC = 0.68, + pre-arrest admission known during admission AUC = 0.73	n.r.	Internal validation: split-sample
Danciu et al. [25]	Pre + intra-arrest	1, 2 3: Respiratory arrest, initial rhythm, chronic renal insufficiency, higher BMI, less days from admission to resuscitation attempt	Scoring system + logistic model equation	1. GOF* p* = 0.874	n.r.	Survival to discharge: sensitivity= 0.88 specificity = 0.32	n.r.
				2. GOF* p* = 0.599		1 month: sensitivity = 0.89 specificity = 0.31	
				3. GOF* p* = 0.822		3 months: sensitivity = 0.91 specificity = 0.32	
Cooper et al. [26]	Pre + intra-arrest	Age, initial rhythm, primary cardiac/respiratory arrest	Scoring system	n.r.	n.r.	Accuracy 90% for 24 h survival for cases lasting > 15 min	n.r.
Ambery et al. [27]	Pre-arrest	Cardiac history, COPD/asthma/respiratory failure, stroke, malignancy, renal insufficiency	Scoring system	n.r.	n.r.	(score of > 4) Under 75y sensitivity 83%, specificity 100%. Over 75y sensitivity 40%, specificity 85%, all sensitivity 52%, specificity 93%	n.r.
Dodek et al. [28]	Pre + intra-arrest	Higher probability of death: Age, female gender, no. Previous arrests, electrical–mechanical dissociation. Lower: underlying coronary artery disease/valvular heart disease, VT, period July–September	Estimates from logistic model	n.r.	AUC:* D* 0.81,* V*: 0.71	*D*: senitivitys + specificity: 0.75 (cut-off probability 0.75);* V*: sensitivity + specificity: 0.6 (cut-off probability 0.85)	External validation: temporal validation
Ebell et al. [29]	Pre-arrest	Age, sex, heart rate, respiratory rate, FiO2, reason for admission, cancer, acute renal failure, GCS, place of residence before admission, mode of transport to hospital, white blood count, sodium, potassium, creatinine, haematocrit, temperature, MAP, pH, and others	Neural network	n.r.	AUC: 0.765 (SE = 0.048)	Sensitivity 52.1%, PPV 97%	n.r.
Lawrence et al. [30]	Pre-arrest	Shock, abnormal BUN, abnormal PaO2, oliguria	Scoring system	n.r.	n.r.	Sensitivity 76%, specificity 65%	n.r.
Marwick et al. [31]	Pre + intra-arrest	1.Age, initial rhythm, CPR delay, defibrillation delay	Scoring system + regression coefficients	n.r.	AUC = 0.78	n.r.	n.r.
		2.Age, initial rhythm, CPR delay			AUC = 0.71		
		3.Age, initial rhythm, defibrillation delay, defibrillated, intubated			AUC = 0.80		
George et al. [32]	Pre-arrest	Hypotension, azotemia, malignancy, pneumonia, homebound lifestyle, angina pectoris, acute MI, heart failure (NYHA III or IV), S3 gallop, oliguria (< 300 ml/day), sepsis, mechanical ventilation, recent cerebrovascular event, coma, cirrhosis	Scoring system + nomogram	n.r.	n.r.	Linear correlation for ROSC:* p* < 0.02; survival to discharge* p* < 0.002; 3 month survival *p* < 0.002. PAM > 7:* p* < 0.0006 for in-hospital mortality	n.r.
Burns et al. [33]	Pre + intra-arrest	Age, surgery scheduled before arrest, intensive care admission pre-arrest, pO2 < 8 mmHg	Scoring system and model equation	n.r.	n.r.	Sensitivity 76%, specificity 61%, accuracy 69%	n.r.
*Model updating*							
Hong et al. [34]	Pre-arrest	GO-FAR + albumin	Scoring system	n.r	AUC D = 0.848 (CI = 0.802–0.893)* V* = 0.799 (CI = 0.745–0.853)	Net reclassification index* V* = 0.072 (CI 0.013–0.132)	External validation: temporal validation
George et al. [35]	Pre-arrest	Age, admission CPC < 2, medical non-cardiac admission, surgical admission, hypotension/hypoperfusion, respiratory insufficiency, septicaemia, metastatic cancer, renal dialysis, hepatic insufficiency	Scoring system	Hosmer-Lemeshow test for calibration 21.43 (*P* = 0.006)	AUC training = 0.70 testing = 0.70 validation = 0.69	Classification table	Internal validation: split-sample
Piscator et al. [36]	Pre-arrest	Neurologically intact admission, sepsis, pneumonia, hypotension, respiratory insufficiency, medical non-cardiac admission, acute kidney injury, CCI, age	Scoring system	Calibration plot	*V*: AUC = 0.808 (CI 0.807–0.810)	For likelihood > 3% sensitivity = 99.4%, specificity = 8.4%	Internal validation: bootstrapping
Dautzenberg et al. [37]*	Pre-arrest	Hypotension, uremia, malignancy, pneumonia, homebound lifestyle, angina pectoris, acute MI after 2 days, heart failure (NYHA III or IV), S3 gallop, oliguria (< 300 ml/day), sepsis, mechanical ventilation, recent cerebrovascular event, coma, age, dementia	Scoring system	n.r	n.r	n.r	n.r
Ebell et al. [38]*	Pre-arrest	Malignancy (metastatic, non-metastatic), sepsis, dependent lifestyle, pneumonia, creatinine > 130 µmol/L, age > 70, acute MI (higher survival)	Scoring system	n.r	n.r	n.r	n.r

*Studies do not formally fit the inclusion criteria due to lack of reporting model performance measures. They were nevertheless included as they were validated in external populations. They validated the scores for different end points but no formal process of model development has taken place. *D* Derivation,* V* Validation  *n.r*. not reported

### Model development and updating studies

The most frequently predicted outcome was survival to discharge (11 studies), followed by ROSC (8 studies), as shown in Table 1. Survival to discharge with a CPC of 1 (2 studies) [21, 22] or ≤ 2 (3 studies) [34–36] was also reported as predicted outcome. Two studies included 3-months survival in their outcomes [25, 32]. Sample size varied from 122 to 92.706 patients. The model updating studies either updated the Good Outcome Following Attempted Resuscitation (GO-FAR) Score (*n* = 3) [34–36] or the Pre-Arrest Morbidity (PAM) Index (*n* = 2) [37, 38].

Of the model development studies, 10 included pre- and intra-arrest factors and six exclusively pre-arrest factors (Table 2). All model updating studies only included pre-arrest factors, according to the models they were based on. A tabular overview is provided of the most frequently included pre-arrest factors affecting clinical outcome after attempted resuscitation: age, dependent functional status, (metastatic) malignancy, heart disease, cerebrovascular event, respiratory, renal or hepatic insufficiency, hypotension and sepsis (Table 3) (a full overview of the parameters per model is included in the Additional file 1: Appendix).

**Table 3:** Overview of included predictors

	Model development																Model updating				
	Swindell [18]	Chan [19]	Harrison [20	Ebell [21]	Ebell [21] CART	Chan [23]	Larkin [24]	Danciu [25]	Cooper [26]	Ambery [27]	Dodek [28]	Ebell [29]	Lawrence [30]	Marwick [31]	George [32]	Burns [33]	Hong [34]	George [35]	Piscator [36]	Dautzenberg [37]	Ebell [38]
*Pre-admission factors*																					
Age	x	x	x	x	x	x	x		x		x	x		x		x	x	x	x	x	x
Sex			x								x	x									
Dependent functional status (or CPC 2)				x	x		x					x			x			x		x	x
(Metastatic/haematological) malignancy	x	x		x		x	x			x		x			x		x	x		x	x
Charlson Comorbidity Index																			x		
Chronic renal insufficiency								x		x								x			
Hepatic insufficiency/cirrhosis	x																	x			
*(Post-)admission factors*																					
Respiratory insufficiency		x		x								x					x	x	x		
Renal insufficiency				x	x		x					x	x		x		x		x	x	x
Hepatic insufficiency/cirrhosis				x	x	x	x								x		x				
Sepsis				x	x	x	x								x		x	x	x	x	x
Heart failure/heart disease/AP		x					x								x					x	x
Cerebrovascular event				x	x		x								x		x			x	
Hypotension/IV vasopressor		x		x		x	x					x	x		x		x	x	x	x	
Mechanical ventilation		x			x	x	x								x					x	
Peri-arrest factors*																					
Initial rhythm		x	x			x	x	x	x		x			x							
Days from admission to arrest	x		x				x	x													
Location of arrest	x	x	x						x	x											

*As this review only includes models that were developed with mainly pre-arrest factors, this section represents only reported peri-arrest factors within these models and will not be a complete overview

Half of the developed/updated models were validated in the same paper either by split-sample (internal) validation [19, 22–24, 35] or temporal (external) validation [20, 21, 28, 34]. In one study, a bootstrapping technique was used [36]. For the remaining 11 studies, no internal validation or recalibration had taken place [18, 25–27, 29–33, 37, 38].

Formally, part of the exclusion criteria was the absence of performance measures, but as the Modified PAM Index (MPI) [37] and Prognosis After Resuscitation (PAR) [38] are frequently validated externally, these studies were included with a disclaimer to the overview for this purpose.

### Model validation studies

In the 12 model validation studies, a total of seven risk models were independently validated in external populations (Table 4): the GO-FAR score [22], the PAM Index [32], the PAR score [38], the MPI [37], two classification and regression tree models (CARTI and CARTII) [21] and the APACHE III score [47]. The most frequently externally validated models are the PAM (*n* = 6) [45–50], GO-FAR (*n* = 5) [39–42, 44], and PAR (*n* = 5) [45–48, 50]. The source of data was most frequently a retrospective cohort (*n* = 10) and twice [40, 42] registry data were used. Sample size varied from 86 to 62.131 patients. In six instances, the validation study was fully independent, meaning the authors of initial score were not implicated in the validation study [39, 41, 45, 46, 49, 50]. There does not seem to be a difference between reported validation performance of the fully independent validation studies and the other external validation studies. Calibration performance was reported in two studies [40, 42]. Area under the receiver operating characteristic curve estimates was calculated in 10 validation studies.

**Table 4:** Characteristics of model validation studies

Reference (year of publication)	Model validated	Study population (cohort type, included patients)	No. of participants (no. with predicted outcome)	Study interval	Source of data	Country of inclusion (no. of centres)	Predicted outcome	Method of validation	Evaluation of model performance		
									Calibration	Discrimination	Classification
Cho et al. [39]	GO-FAR	Attempted CPR for IHCA	1011 (162)	2013–2017	Retrospective cohort	South Korea (1)	Survival to discharge with CPC 1 or 2	Fully independent validation	n.r.	AUC: 0.81(CI 0.78–0.84)	Best at 4 for poor outcome: sensitivity 72.5% (CI 69.5–75-5), specificity 78.6% (CI 70.4–85.4), PPV 96.0% (CI 94.4–97.1), NPV 29.0% (CI 26.1–31.9)
Thai et al. [40]	GO-FAR	Attempted CPR for IHCA	62.131 (9347)	2010–2016	GWTG Registry	USA (386)	Survival to discharge with CPC = 1	External validation; authors include initial author of score	Calibration plot (score systematically underestimates the likelihood of survival with CPC 1)	AUC: 0.75 (CI 0.748–0.758)	Classification table
Rubins et al. [41]	GO-FAR	Attempted CPR for IHCA	403 (133, 70)	2009–2018	Retrospective cohort	USA (1)	Survival to discharge and survival to discharge with CPC = 1	Fully independent validation	n.r.	For survival to discharge with CPC = 1: AUC = 0.68(CI 0.62–0.73)	n.r.
Piscator et al. [42]	GO-FAR	Attempted CPR for IHCA	528 (141) (complete cases)	2013–2014	SCR Registry	Sweden (6)	Survival to discharge with CPC = 1	External validation; authors include initial author of score	Calibration plot (GO-FAR model under-predicts the probability of survival with CPC = 1)	Complete cases: AUC = 0.82(CI 0.78–0.86) (AUC = 0.80 (CI 0.76–0.84) for multiple imputation analysis)	Classification table
Limpawattama et al. [50]	PAM, PAR, MPI	Attempted CPR for IHCA	278 (72) (166 complete IHCA cases)	2013–2014	Retrospective cohort	Thailand (1)	Survival to discharge	Fully independent validation	n.r.	AUC: PAM = 0.62(CI 0.51–0.73), PAR = 0.60(CI 0.49–0.71), MPI = 0.63(CI 0.52–0.73)	n.r. for only IHCA
Guilbault et al. [43]	CART1, CART2	Attempted CPR for IHCA	287 (45)	2007–2010	Retrospective cohort	Sweden (1)	Survival to discharge with CPC = 1	External validation; authors include initial author of score	n.r.	CART 1: AUC = 0.77. CART 2: AUC = 0.71	Classification table
Ohlsson et al. [44]	GO-FAR	Attempted CPR for IHCA	287 (45)	2007–2010	Retrospective cohort	Sweden (1)	Survival to discharge with CPC = 1	External validation; authors include initial author of score	n.r.	AUC: 0.85 (CI 0.78–0.91)	Best sensitivity and specificity at -3.5: 79% and 78%
Ohlsson et al. [45]	PAM, PAR	Attempted CPR for IHCA	287 (58)	2007–2010	Retrospective cohort	Sweden (1)	Survival to discharge	Fully independent validation	n.r.	AUC: PAM = 0.601(CI 0.528–0.674 *p* = 0.018), PAR = 0.718(CI 0.647–0.79 *p* = < 0.001)	Sensitivity, specificity: PAM > 5 = 21.8%, 94.8%. PAR > 5 = 31%, 94.8%
Bowker et al. [46]	PAM, PAR, MPI	Attempted CPR for IHCA	264 (28)	1994–1996	Retrospective cohort	UK (1)	Survival to discharge	Fully independent validation	n.r.	n.r	Sensitivity: PAM > 6 = 20%, PAR > 7 = 29%, MPI > 6 = 22% (specificity for all 100%)
Ebell et al. [51]	PAM, PAR, APACHE III	Attempted CPR for IHCA	656 (248, 35)	2–3 years (not further specified)	Retrospective cohort	USA (3)	ROSC with transfer to ICU & survival to discharge	External validation; authors include initial author of score	n.r.	AUC: ROSC: PAR = 0.51(SE = 0.023), PAM = 0.495(SE = 0.023), APACHE III 0.495(SE = 0.023). Survival to discharge: PAR = 0.564(SE = 0.05), PAM = 0.519(SE = 0.051), APACHE III = 0.587(SE = 0.049)	n.r.
O'keeffe et al. [48]	PAM, PAR	Attempted CPR for IHCA	274 (25)	2 years (not further specified)	Retrospective cohort	Ireland (1)	Survival to discharge	External validation; authors include initial author of score	n.r.	AUC: PAM = 0.67 (SE = 0.06) PAR = 0.74 (0.05)	Sensitivity: PAM > 4 = 9.2%, > 8 = 2%. PAR: > 5 = 23.7%, > 8 = 9.6%. Specificity: 100% for both scores at all cut-off points
Cohn et al. [49]	PAM (minus S3 gallop)	Survivors of attempted CPR for IHCA and matched non-survivors	86 (43)	1986–1991	Retrospective case–control	USA (1)	Survival to discharge	Fully independent validation	n.r.	*p* < 0.0003; *R*^2^ = 9.6%	n.r.

### Meta-analysis

**Fig. 2:**
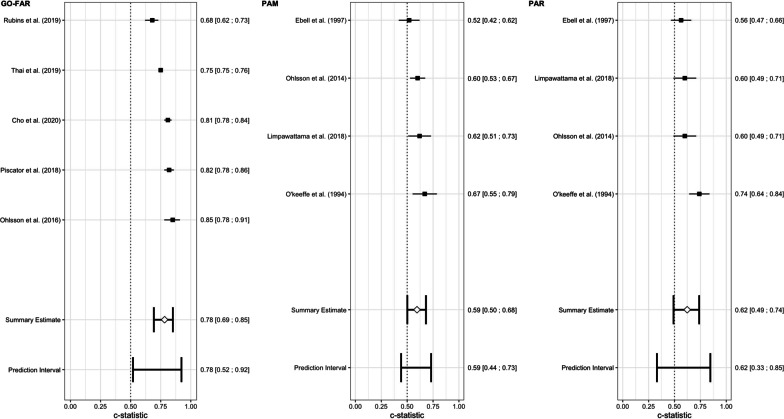
Forest plots of c-statistics in external validation studies

It was possible to calculate a pooled performance of the GO-FAR [39–42, 44], PAM [45, 47, 48, 50] and PAR [45, 48, 50, 51] scores (Fig. 2. Forest plots of c-statistics in external validation studies.). The GO-FAR score showed the best performance with a pooled AUROC of 0.78 (95% CI 0.69–0.85), versus 0.59 (95%CI 0.50–0.68) for the PAM and 0.62 (95%CI 0.49–0.74) for the PAR.

### PROBAST

The assessment of quality with the PROBAST tool showed most risk of bias was present in the ‘analysis’ domain (full assessment in the Additional file 1: Appendix): the number of participants was not always satisfactory, and frequently the way in which missing data were handled was not reported.

## Discussion

This systematic review describes prognostic models that use pre-arrest factors to predict outcome of in-hospital cardiac arrest. A comprehensive overview of developed, updated and validated models is presented. Using the best available evidence, i.e. the best performing model, could aid patients and clinicians in making an informed decision whether to attempt or refrain from CPR. Only six models have been validated in external populations. Of these, the GO-FAR score shows the most acceptable performance.

### Model development and updating

This systematic review shows that there has been a plethora of prognostic models developed in recent years to predict outcome after IHCA. A total of 27 different prognostic models were published using pre-arrest factors to predict any clinical outcome after IHCA. Approximately half use pre- and intra-arrest factors and the remaining half exclusively pre-arrest factors, which are the models would be more useful in a clinical setting. However, the time at which the factors are assessed often differs from the moment at which the model would be used; as is illustrated by the validation study of the GO-FAR score from Rubins et al. [41]. The authors found the lowest AUC of the GO-FAR score when using it with admission factors, instead of data collected close to the IHCA, which is to be expected as the score was not developed for this moment. However, this demonstrates a potential pitfall of the prognostic models if used in clinical practice. The clinical course of a patient admitted to the hospital is a dynamic process, which in an ideal situation the models would reflect: initially only including pre-arrest factors known at admission and gradually incorporating peri-arrest factors as the clinical situation evolves. A potential problem of prognostic models including both pre-arrest factors and peri-arrest factors is that the peri-arrest factors carry a lot of weight in the model but they are not known at the time of initial counselling. Their importance becomes evident in later clinical decision-making, when deciding whether to (dis)continue a resuscitation attempt. The impact of peri-arrest variables on outcome should be reflected in decision models for termination of CPR, whereas the pre-arrest variables studied in this review should allow for better patient counselling on advance care directives.

As Lauridsen et al. rightfully note, in a recent review of test accuracy studies for IHCA prognosis, there is a need for models that aid in prognostication at an early stage of hospital admission, so that patients (and/or family) can be properly informed. They concluded that no score was sufficiently reliable to support its use in clinical practice. Our study provides a comprehensive review of model development, updating and validating, rather than just the diagnostic accuracy of the tools where no distinction between model development and validation is made [6]. We have critically appraised the methodology behind each model using the PROBAST—as is appropriate in this instance—and have managed to perform a meta-analysis of the models’ performances, using methodological guidance on meta-analysis of prediction model performance [8]. We did specifically not include Early Warning Scores, as they are comprised of physiological parameters that are not available at the time on counselling and are used to estimate the risk of deterioration in hospitalised patients rather than the prognosis after IHCA. They proved to be highly inaccurate for prediction of patient survival. Excluding the studies investigating Early Warning Scores, Lauridsen et al. included 20 studies, whereas this systematic review includes 33 studies maybe due to a search strategy more specific for a systematic review of prediction models [10].

Age was included in almost all models as a prognostic factor for outcome after IHCA. Dependent functional status was also a frequently included factor, as were comorbidities (metastatic) malignancy, renal insufficiency and the presence of sepsis. This corresponds with findings of a recent systematic review evaluating the association of single pre-arrest and intra-arrest factors with survival after IHCA, where the pre-arrest factors age, active malignancy and chronic kidney disease were all independently associated with reduced survival [52]. Male sex was also found to be an independent prognostic factor, but it was only in three of the models included in this systematic review. Frailty has recently been found to be a robust prognostic factor for in-hospital mortality after IHCA, which is reflected in this study by dependent functional status or admission from a nursing facility being frequently included prognostic factors [53]. It is however debateable whether frailty and functional dependence are the same thing. This was recently demonstrated in an observational study, where it was found that moderately frail adults demonstrate heterogeneity in functional status [54].

A wide diversity of predicted outcomes is present in the included models, ranging from the occurrence of ROSC to survival to discharge with a good neurological outcome. And although CPC is not a patient-centred outcome measure, it does provide an extra dimension over survival. Given that the GO-FAR performance is still better than other models, future research should attempt to correlate this model’s variables to health-related quality of life (HRQoL). And as previously argued by Haywood et al., all future cardiac arrest research should use uniform reporting of long-term outcomes and HRQoL to allow for better comparison between studies and represent more clinically relevant outcomes [55–57].

### Model validation

To maximize the potential and clinical usefulness of prognostic models, they must be rigorously developed and—internally and externally—validated, and their impact on clinical practice and patient outcomes must be evaluated. Model development studies should adjust for overfitting by performing internal validation and recalibration. Several techniques for internal validation (reproducibility) are used and include apparent validation (development and validation in the same population), split-sample validation (random division of data in training and test sets) and bootstrapping (random samples of the same size are drawn with replacement). Only half of the studies in this systematic review which developed scores engaged in some form of (mainly internal and split-sample) validation.

However, no score should be applied in clinical settings unless it has been externally validated. External validation (generalisability) of a model can be performed via geographical or temporal validation or a fully independent validation (with other researchers at another centre) [14]. Only six models were subsequently validated in external populations and only a minority of the models assessed calibration or mention recalibration of the presented model. This could mean an overall overestimation of the performance of the other reported prognostic models. Performance is easily overestimated when there is only apparent validation. Therefore, external validation studies are needed to ensure the generalisability of a prognostic model for medical practice [58]. Moreover, only a minority of the models assessed calibration or mention recalibration of the presented model.

Based on the prognostic models identified through this systematic review, the GO-FAR score has the best performance when validated in external populations and is at this time the most robust and tested model. The performance of the PAM, PAR and MPI in external validation studies limits its consideration for clinical use.

As for generalisability; models were predominantly developed in the USA (using GWTG Registry data) and the UK. Several external validation studies were performed in Sweden in the same relatively small retrospective cohort. This emphasises a need for external model validation and updating in different populations, as many countries are not represented in the current body of literature and important cultural differences play an important role in the installing of advance care directives [59].

### Strengths and limitations

This study contains a comprehensive search and extensive analysis using current guidelines for reviewing and assessing bias of prediction model studies [7, 17]. Methodological assessment revealed that the most frequent risk of bias was introduced in the domains source of data, sample size, number of outcomes and analysis (Additional file 1: Appendix.) Limitations pertain mainly to design of the included studies. Only two models were developed with prospective collected data, as is reported to be the superior source of data for the development of prognostic models [13, 17]. Most models were developed using registry data or relatively small retrospective cohorts. Another limitation of this study includes low sensitivity of the search, due to a lack of search terms and indexing for prognostic model studies [10].

### Methodological recommendations

An important caveat in interpreting these results and implementing them  in practice becomes apparent when examining the prognostic models as the time at which the factors are assessed often differs from the moment at which the model would be used in a clinical setting. A prognostic model meant to be used before starting CPR (at hospital admission, or even prior to that moment) might be more practical and better reflect the moment when the decision-making in advance care planning is taking place and when such a model could be most helpful.

Imputation techniques should be used when data are missing and the full equation of the prognostic model should be presented to allow for external validation and updating by independent research teams and this should be performed in large prospective cohorts. Calibration is an important aspect of performance and should be assessed in future studies, as poorly calibrated models can be unreliable even with good discrimination [16].

There seems to be a gap between the development of prognostic models and the researching of their possible effect on clinical decision-making and maybe even on patient outcomes. Furthermore, clinicians may be eschewing the use of scores due to lack of clear guidance on which score(s) to use, barriers to practical use, or they may find the utility of the scores limited in clinical practice. In spite of the prevalence of risk models, it is known few models have been validated, and even fewer are used regularly in clinical settings [58, 60]. Future research should focus on updating and validating existing prediction models in large external populations, rather than developing new models. After extensive external validation studies of prognostic models, implementation studies are needed to assess their influence in clinical practice [61].

## Conclusions

Several prediction models for clinical outcome after attempted resuscitation for IHCA have been published, most have a moderate risk of bias and have not been validated externally. The GO-FAR-score is the only prognostic model included in multiple external validation studies with a decent performance. Future research should focus on updating existing models in large external populations and on their influence on clinical decision-making.

## Supplementary Information

**Additional file 1.** Supplementary materials: search strategy and PROBAST assessment.

## Data Availability

The dataset used for the meta-analysis is available from the corresponding author upon reasonable request, all other data generated of analysed during this study are included in this published article and its supplementary files.
