# Supplementary material for: Prognostic models for outcome prediction following in-hospital cardiac arrest using pre-arrest factors: a systematic review, meta-analysis and critical appraisal
Source: Crit Care. 2023 Jan 20;27:32. doi: 10.1186/s13054-023-04306-y (PMC9862512; doi:10.1186/s13054-023-04306-y)
Supplement: Supplementary file 1 — Additional file 1. Supplementary materials: search strategy and PROBAST assessment. [file 13054_2023_4306_MOESM1_ESM.docx]

***Supplementary material***

| **PICOTS** |  |
| --- | --- |
| Population | Adult patients in whom conventional cardiopulmonary resuscitation for in-hospital cardiac arrest is attempted |
| Intervention (model) | Prognostic model to predict any possible clinical outcome after IHCA, with pre-arrest factors, or pre- and intra-arrest factors |
| Comparator | Not applicable |
| Outcome | Any clinical outcome after IHCA |
| Timing | Predictors measured before IHCA and therefore available for the clinician at the time of advance care planning; outcome measured in short term or long term |
| Setting | Admitted patients to hospital wards, monitored wards, ICU or emergency department |

**Table 1. PICOTS of review question**

**Literature search**

| **Database searched** | **via** | **Years of coverage** | **Records** | **Records after duplicates removed** |
| --- | --- | --- | --- | --- |
| Embase | Embase.com | 1971 - Present | 2095 | 2076 |
| Medline ALL | Ovid | 1946 - Present | 1048 | 558 |
| Web of Science Core Collection* | Web of Knowledge | 1975 - Present | 414 | 44 |
| **Total** | | | **3557** | **2678** |

*Science Citation Index Expanded (1975-present) ; Social Sciences Citation Index (1975-present) ; Arts & Humanities Citation Index (1975-present) ; Conference Proceedings Citation Index- Science (1990-present) ; Conference Proceedings Citation Index- Social Science & Humanities (1990-present) ; Emerging Sources Citation Index (2015-present)

**Embase**

((('heart arrest'/de OR 'cardiopulmonary arrest'/de OR 'resuscitation'/de) AND ('hospital patient'/de OR 'aged hospital patient'/de OR hospitalization/de OR 'hospital discharge'/de OR 'hospital mortality'/de)) OR 'in hospital cardiac arrest'/de OR (((in-hospital OR inhospital OR inpatient* OR hospitali* OR hospital-discharg* OR patient-discharg*) NEAR/6 (cardi*-arrest* OR resuscitat* OR CPR)) OR IHCA):Ab,ti,kw) AND ('prognostic model'/de OR ((prognosis/de OR survival/de OR 'survival rate'/de OR resuscitation/de OR circulation/de OR 'return of spontaneous circulation'/de OR prediction/de OR 'predictive value'/de OR 'predictor variable'/de OR 'prediction and forecasting'/de OR 'outcome assessment'/de OR probability/de) AND (model/de OR 'machine learning'/exp OR 'artificial intelligence'/exp OR algorithm/exp OR 'regression analysis'/exp OR 'scoring system'/de OR 'validation process'/de OR 'decision tree'/de)) OR ((prognosis/de OR survival/de OR 'survival rate'/de OR 'outcome assessment'/de) AND (prediction/de OR 'predictive value'/de OR 'predictor variable'/de OR 'prediction and forecasting'/de OR probability/de)) OR 'survival prediction'/de OR (((prognos* OR surviv* OR outcome* OR resuscitation* OR circulat* OR predict*) NEAR/3 (model* OR machine-learning OR artificial*-intelligen* OR algorithm* OR logistic-regression* OR regression-analys* OR scoring-system*)) OR ((predict*) NEAR/3 (prognos* OR surviv* OR outcome*)) OR ((prognosis) NEAR/3 (resuscitation OR cardi*-arrest*)) OR PAR):ab,ti,kw) NOT ((juvenile/exp OR pediatrics/exp) NOT (adult/exp)) NOT ([conference abstract]/lim AND [2000-2018]/py)

**Medline ALL Ovid**

(((Heart Arrest / OR exp Resuscitation/) AND (Inpatients / OR Hospitalization / OR Hospital Mortality /)) OR (((in-hospital OR inhospital OR inpatient* OR hospitali* OR hospital-discharg* OR patient-discharg*) ADJ6 (cardi*-arrest* OR resuscitat* OR CPR)) OR IHCA ).ab,ti,kf.) AND (((Prognosis/ OR Survival/ OR Survival Rate/ OR Resuscitation/ OR Blood Circulation/ OR Return of Spontaneous Circulation/ OR Predictive Value of Tests/ OR Forecasting/ OR Outcome Assessment, Health Care/ OR Probability/) AND (Models, Statistical / OR exp Artificial Intelligence / OR Algorithms / OR Regression Analysis / OR Validation Study / OR Decision Trees /)) OR ((Prognosis/ OR Survival/ OR Survival Rate/ OR Outcome Assessment, Health Care /) AND (Predictive Value of Tests/ OR Forecasting / OR Probability /)) OR survival prediction/ OR (((prognos* OR surviv* OR outcome* OR resuscitation* OR circulat* OR predict*) ADJ3 (model* OR machine-learning OR artificial*-intelligen* OR algorithm* OR logistic-regression* OR regression-analys* OR scoring-system*)) OR ((predict*) ADJ3 (prognos* OR surviv* OR outcome*)) OR ((prognosis) ADJ3 (resuscitation OR cardi*-arrest*)) OR PAR).ab,ti,kf.) NOT ((exp child/ OR exp infant/ OR adolescent/ OR pediatrics/) NOT (exp adult/))

**Web of Science Core Collection**

TS=((((in-hospital OR inhospital OR inpatient* OR hospitali* OR hospital-discharg* OR patient-discharg*) NEAR/5 (cardi*-arrest* OR resuscitat* OR CPR)) OR IHCA) AND ((((prognos* OR surviv* OR outcome* OR resuscitation* OR circulat* OR predict*) NEAR/2 (model* OR machine-learning OR artificial*-intelligen* OR algorithm* OR logistic-regression* OR regression-analys* OR scoring-system*)) OR ((predict*) NEAR/2 (prognos* OR surviv* OR outcome*)) OR ((prognosis) NEAR/2 (resuscitation OR cardi*-arrest*)) OR PAR))) AND DT=(Article OR Review OR Letter OR Early Access)

| Study | ROB | | | | Applicability | | | Overall | |
| --- | --- | --- | --- | --- | --- | --- | --- | --- | --- |
|  | Participants | Predictors | Outcome | Analysis | Participants | Predictors | Outcome | ROB | Applicability |
| Burns (1989) | + | ? | + | - | + | + | - | - | - |
| George (1989) | + | ? | + | - | + | + | + | - | + |
| Marwick (1991) | + | ? | + | - | + | + | + | - | + |
| Lawrence (1991) | + | ? | + | - | + | + | + | - | + |
| Ebell (1993) | + | ? | + | - | + | + | + | - | + |
| Dodek (1998) | + | - | + | - | + | + | + | - | + |
| Ambery (2000) | + | + | + | - | + | + | + | - | + |
| Cooper (2003) | + | + | + | - | + | + | + | - | + |
| Danciu (2004) | + | ? | + | - | + | + | + | - | + |
| Larkin (2010) | - | + | + | + | + | + | + | - | + |
| Chan (2013) | - | + | + | + | + | + | + | - | + |
| Ebell (2013) (GO-FAR) | - | + | + | + | + | + | + | - | + |
| Ebell (2013) | - | + | + | + | + | + | + | - | + |
| Harisson (2014) | - | + | + | + | + | + | + | - | + |
| Chan (2020) | - | + | + | + | + | + | - | - | - |
| Swindell (2021) | - | + | + | - | - | + | + | - | - |
|  |  |  |  |  |  |  |  |  |  |
| Ebell (1992) | - | ? | ? | ? | ? | + | + | - | ? |
| Dautzenberg (1993) | - | - | ? | - | ? | + | + | - | ? |
| Piscator (2019) | - | + | + | - | + | + | + | - | + |
| George (2020) | - | + | + | - | + | + | + | - | + |
| Hong (2021) | + | + | + | - | + | + | + | - | + |

**PROBAST Appraisal of model development and update studies.**

| Study | ROB | | | | Applicability | | | Overall | |
| --- | --- | --- | --- | --- | --- | --- | --- | --- | --- |
|  | Participants | Predictors | Outcome | Analysis | Participants | Predictors | Outcome | ROB | Applicability |
| Cohn (1993) | + | + | + | - | + | + | + | - | + |
| O'Keeffe (1994) | + | + | + | - | + | + | + | - | + |
| Ebell (1997) | + | + | + | - | + | + | + | - | + |
| Bowker (1999) | + | + | + | - | + | + | + | - | + |
| Ohlsson (2014) | + | + | + | - | + | + | + | - | + |
| Ohlsson (2016) | + | + | - | - | + | + | + | - | + |
| Guilbault (2017) | + | + | + | - | + | + | + | - | + |
| Limpawattama (2018) | - | + | + | - | - | + | + | - | - |
| Piscator (2018) | - | + | + | + | + | + | + | - | + |
| Rubins (2019) | + | + | + | - | + | + | + | - | + |
| Thai (2019) | - | + | + | + | + | + | + | - | + |
| Cho (2020) | + | + | + | + | + | + | + | + | + |

**PROBAST Appraisal of validation studies.**
